# *Proceedings B* 2024: the year in review

**DOI:** 10.1098/rspb.2025.0065

**Published:** 2025-01-29

**Authors:** Spencer C. H. Barrett

**Affiliations:** ^1^ Editor-in-Chief

It has been a successful year at *Proceedings B* with increased submissions and by year’s end we surpassed our target and received 3006 submissions. The two main initiatives that we introduced this year—double anonymous peer review and the use of data editors—have both been successful and are now fully implemented in the review process. From 1 January to 31 October 2024, we received 2546 submissions, an increase of 178 papers compared with the same period in 2023 and around 5% higher than in 2023. Our rejection rate was 79%, somewhat higher than in the previous few years. Our times (in median days) to first decision improved to 17 compared with 19 in 2023, although days to final decision (77) and publication (115) increased because of the time required for data checks and implementation of double anonymous peer review. Also, the move to a new typesetter—Kriyadocs—also resulted in longer times to online publication during the implementation phase. So far, 304 papers were published open access, an increase of 25 papers compared with the same period in 2023 and open access content now represents 63% of all papers that we publish. In common with other journals publishing similar content, our impact factor dropped from 4.7 to 3.8. *Proceedings B* is currently ranked 18 out of 109 journals in the Journal Citation Reports category ‘Biology’. Ecology, evolution and behaviour remain the most popular subject areas for submission and publication.

*Proceedings B* is a truly international journal receiving submissions from diverse geographical regions, with the largest numbers (1 January–31 October) coming from the USA (614), China (389), UK (268), Australia (115), Germany (138), France (111), Canada (98), Japan (98), Spain (63) and Sweden (49). We continue our efforts to encourage submissions from under-represented regions and recruiting new Associate Editors from these areas can aid in this endeavour. In this year’s round of new recruits, 17% of new Associate Editors who start their terms in 2025 are from countries in Asia, Africa and South America. In addition, as discussed below, this year we initiated a new Special Feature designed to highlight researchers from the Global South and indigenous communities, and we hope that this will lead to more submissions from under-represented regions and demographic groups.

*Proceedings B* reviews provide an outstanding forum for timely treatment of emerging areas and unresolved questions in biology and are well cited. In 2024, we said goodbye to Innes Cuthill (University of Bristol), who for the past seven years has been the Reviews Editor for *Proceedings B* and prior to that a senior editor for the journal. I know I speak for all editors and staff in thanking Innes for his dedication and hard work in maintaining a steady stream of high-quality reviews in the journal. The journal received fewer Review proposals in 2024, with 64 compared to 74 in 2023. By the end of October, 16 Reviews were published as compared to 15 in 2023. Those interested in writing a review for *Proceedings B* should download the proposal form from our website https://royalsocietypublishing.org/rspb/reviews and submit it to the journal for consideration.

During the summer, we recruited a new Reviews Editor—Joel McGlothlin—from Virginia Tech University in the USA. Joel is an evolutionary biologist interested in how natural selection influences the evolution and maintenance of complex traits. His research integrates data from evolutionary genetics, behavioural ecology and physiological ecology and he has worked on a wide range of systems including birds, lizards, snakes and plants. Joel is originally from Tennessee and graduated from Vanderbilt University in 2001 where he worked with the late Dave McCauley. He then conducted PhD research at Indiana University advised by Ellen Ketterson, where he investigated how multivariate phenotypes evolve, graduating in 2007. After a postdoctoral fellowship at the University of Virginia working with Butch Brodie on the evolutionary quantitative genetics of lizards, the evolution of toxin resistance in snakes and the theory on how social interactions influence evolution, Joel moved in 2012 to the Department of Biological Sciences at Virginia. Joel won the 2011 Dobzhansky Prize awarded by the *Society for the Study of Evolution* and is a former Editor and Secretary of *The American Society of Naturalists*.

The annual Darwin review, ‘Pollination ecotypes and the origin of plant species’ [1] by Steven Johnson (University of KwaZulu-Natal) is now formally accepted in *Proceedings B* and will be published shortly. The article reviews evidence that populations characterized by discrete patterns of intraspecific genetically-based floral variation, often referred to as ecological races or ‘ecotypes’, can often be a prelude to speciation. The ecotype concept, which has a long and venerable history in plants [2], has most commonly been investigated as responses to abiotic selection along environmental gradients or in soil mosaics. Numerous examples of climatic and edaphic ecotypes are reported in the plant literature, and reciprocal transplant studies have commonly been used to demonstrate the adaptive basis of ecotypic differentiation. In his article, Johnson provides compelling evidence that biotic selection imposed by local pollinators can result in microevolutionary responses and novel floral trait combinations in geographically structured populations. Such divergent selection resulting in pollination ecotypes can lead to cases of species *in statu nascendi*. Johnson breaks with tradition and expands the concept of pollination ecotypes to include cases in which outcrossing mediated by pollinators has been abandoned altogether and replaced by self-pollination and the evolution of the selfing syndrome [3]. Johnson reviews critical practical and conceptual issues for studies of ecotype formation including the paucity of reliable natural history data, the challenges of collecting pollinator information in reciprocal transplant experiments, and deciding when allopatric ecotypes should be considered separate species.

In 2024, we introduced a second named review the ‘Georgina Mace Review’, in honour of the late Professor Georgina Mace FRS, highlighting current topics in conservation and biodiversity science https://royalsociety.org/blog/2024/12/introducing-proceedings-b-inaugural-georgina-mace-review-in-conservation-biology/. Eleanor Milner-Gulland, Director of the Interdisciplinary Centre for Conservation Science at the University of Oxford, and colleagues have written the inaugural review—‘What is a unit of nature? Measurement challenges in the emerging biodiversity credit market’—which was recently published in *Proceedings B* [4]. In the article the authors, who are predominately early career researchers coming from diverse backgrounds including ecology, economics and finance, grapple with the challenging problem of coming up with standardized measurements of biodiversity or what can be considered ‘units of nature.’ Using biodiversity metrics is increasingly recognized by many in the conservation biology community as necessary to enable the business and financial sectors to contribute to funding nature recovery. The article examines how units are defined, presents a general framework on the topic and assesses how to link positive results to investments associated with credit markets. The authors raise critical questions as to whether biodiversity credits and ways of commodifying nature to single units may have unintended consequences. The authors conclude by emphasizing that although there may be a role for markets in generating funds for conservation, such approaches can never be the only solution to conserving biodiversity.

In 2024, the Special Feature ‘The resolution of evolutionary conflicts within species’ edited by J. Arvid Ågren (Case Western Reserve University), Göran Arnqvist (Uppsala University) and Locke Rowe (University of Toronto) was published [5]. In addition, two other Special Features were commissioned and will be published in 2025. The first Special Feature ‘The other 1%: showcasing science and scientists from the Global South and indigenous communities’ edited by Sarah Brosnan (Georgia State University), Stephanie Meirmans (University of Amsterdam), Maurine Neiman (University of Iowa), Guadalupe Peralta (National University of Córdoba) and Shalene Singh-Shepherd (Royal Society Publishing) was initiated following discussions at our board meeting on concerns about the vast overrepresentation of a small number of countries and scientists as sources of authorship in scientific publishing. Indeed, *Proceedings B* is very much part of this disparity, with 75% of submissions in the last five years originating from just 12 countries, all of high income. The Special Feature therefore focuses on the majority of countries that submit 1% or less of the remaining 25% of submissions and will be a compilation of papers that meet a set of criteria outlined on our website: https://royalsocietypublishing.org/rspb/special-features. Most importantly, the research has to fall within the scope of *Proceedings B,* and the primary contributor (first or last author) is from the Global South and/or indigenous communities. By the end of 2024, we received 228 proposals for articles of which 97 have been accepted and the authors have been given the go-ahead to write and submit their papers. Given that most of these authors will not have English as their primary language, and they may not have much experience publishing in broad high-profile journals, the editors of the volume will devote time to mentoring authors through the publication process for those contributions judged to be meritorious.

A second Special Feature initiated in 2024—‘Wildlife behaviour and movement ecology in a human-dominated world’—is being edited by Michael Bertram and Jack Brand (Swedish University of Agricultural Sciences) and Marlee Tucker (Radboud University) and will be published during 2025. This collection of articles is based on the idea that behavioural change is usually the first response of wildlife populations to environmental stressors associated with human activity, and also that technologies and approaches for monitoring wildlife behavioural responses and movement have increased in sophistication in recent years. Articles will include a diverse set of experimental methods, animal taxa and environmental stressors. The Special Feature will provide a focused treatment of the diverse impacts of human-induced rapid environmental change on the behaviour and movement ecology of animal species and should be of interest to behavioural ecologists, wildlife managers and those involved with environmental protection.

Several new journal developments were discussed in my 2023 ‘Year in Review’ [6] and I summarize below progress on each of them. Our online seminar and Q&A channel in ecology and evolution (https://cassyni.com/series/NTMZiEYLfDVdhvAz38B4aW) has now hosted 12 seminars and the feedback we have received is generally positive. Several more seminars are lined up for early 2025 and we are currently in the process of selecting additional speakers from our best performing papers, based on downloads, media attention and Altmetric scores. In January 2024, we rolled out mandatory double anonymous peer review (DAPR) in which the names of authors and reviewers are kept hidden throughout the review process, while editors are able to view author names and affiliations (https://royalsocietypublishing.org/rspb/double-anonymous-peer-review). DAPR has generally received an enthusiastic response from authors, especially among early career researchers. Despite the additional checks on submission that are required in the editorial office to ensure papers are sufficiently anonymized, as indicated above the time to first decision actually improved to 17 days in 2024. Another new initiative that was introduced in 2024 was the addition to our editorial board of eight data editors responsible for checking selected papers at the request of editors handling particular submissions (https://royalsocietypublishing.org/rspb/about#question5). The team of data editors have been working successfully throughout the year, checking an average of 10 papers per month. More information about the individuals who make up the data editor team is available in the following blogs: https://royalsociety.org/blog/2023/12/data-editors-proc-b-part-one/ and https://royalsociety.org/blog/2023/12/data-editors-proc-b-part-two/.

The current landscape for scientific publishing has changed more rapidly over the past two or three decades than at any previous period since the birth of scientific publishing in 1665 [7]. Readers of *Proceedings B* will have noticed numerous changes to the journal during the past decade including transparent open review in which referee’s reports are published along with the article, double anonymous peer review, the use of data editors, communication via blog posts, newsletters and online lectures and the diversification of the editorial board and range of subject areas that we publish. These innovations have all been positive steps in the evolution of the journal. However, at the same time, scientific journals in general have been confronted with a range of new challenges. Many of these are directly linked to the pressures of career advancement in academic and research institutions and the ‘publish or perish’ culture. An overemphasis on the importance of journal impact factors in some research communities has given rise to growing threats to research integrity through data falsification, plagiarism, the misuse of AI, paper mills and even identity theft. Indeed the rise of social media, while having many positive benefits for the rapid communication of scientific discoveries, is also being abused to spread misinformation and feed the claims of the anti-science lobby and some politically motivated media outlets that the peer review process is broken and that many scientists are simply untrustworthy. Of course, as a practising scientist and editor of a journal, I dispute these exaggerated claims while recognizing that reforms are needed.

The Royal Society publishes 10 peer-reviewed journals and therefore it is not surprising that the Publishing Board of the Society takes a strong interest in both current and future developments in scientific publishing. Therefore, to facilitate an in-depth assessment an ‘Advisory Committee on the Future of Scientific Publishing’ was established in 2024 with the goal of exploring a wide range of topics that will influence the nature of science publishing over the next decade. The committee is chaired by Sir Mark Walport FRS, Foreign Secretary and Vice President of the Royal Society, and in addition to the Biological and Physical Secretaries and Director of Publishing of the Royal Society, the committee includes a wide range of expertise comprising editors of journals, representatives from publishing, preprint servers and media outlets, as well as an economist, historian, early career researcher and digital library expert. Committee members work in diverse settings including North America, Australia, Brazil and the UK. This spring, a two-day conference at the Royal Society will review the changing publishing ecosystem and discuss emerging findings and solutions that will guide the society’s future publishing activities as well as all who are involved in scientific publishing. The committee will produce a final report on their findings and propose recommendations during 2025.

I extend thanks to the Senior and Associate Editors of *Proceedings B* for all the work that they have done in handling the large number of submissions that we receive. For those completing their terms and leaving in 2025, I hope that your time with *Proceedings B* has been a valuable experience and that you have benefitted from handling diverse subject areas, some of which you may not have been familiar with previously. Please keep in contact and I hope your careers flourish. I would like to extend my special thanks to Professor Loeske Kruuk FRS who as Senior Editor worked assiduously to maintain the quality of articles in the journal and also took a leadership role in investigating misconduct issues during her tenure. Replacing editors is an annual task as we seek to ensure we are covering key subject areas, but at the same time diversifying the composition of the editorial board. For 2025, we have managed to recruit a talented group of editors. After stints as Associate Editors for the journal, James Bull (Swansea University) and Devi Stuart-Fox (University of Melbourne) take over as Senior Editors. James is a mathematical biologist interested in metapopulations, population dynamics and disease ecology and is studying a variety of systems including honey bees, marine mammals and sea grasses. Devi is an evolutionary and behavioural ecologist who works on the biology of light and colour from the nanometre scale to global patterns of colour diversity. She has worked on diverse animal groups including lizards, honey bees and beetles.

We have recruited 28 new Associate Editors for 2025 of which 48% are female. The list includes: Daniel Anstett (Cornell University), Amanda Bretman (Leeds University), Agnes Dellinger (University of Vienna), Jonathan Drury (Durham University), Zachary Emberts (Oklahoma State University), Ines Fürtbauer (Swansea University), Allison Hsiang (Stockholm University), Susan Johnston (Edinburgh University), Laura Kelley (University of Exeter), Kayla King (University of British Colombia), Amy Krist (Wyoming University), Jussi Lehtonen (University of Jyväskylä), Miguel Lurgi (Swansea University), Ben Longdon (University of Exeter), Paolo Momigliano (University of Hong Kong), Levi Morran (Emory University), Mary Morgan-Richards (Massey University), Kerry Naish (Washington University), Alex Papadopulos (Bangor University), John Ratcliffe (University of Toronto), James Reimer (University of the Ryukyus), Stuart Sandin (UC San Diego), Catherine Searle (Purdue University), Saki Takahashi (John Hopkins University), Maria Tello-Ramos (Hull University), Shunsuke Utsumi (Hokkaido University), Klara Wanelik (University of Surrey) and Eric Wood (California State University). Welcome aboard, and I look forward to meeting some of you at our annual board meeting in London in May.

Finally, I would also like to thank our editorial team in London, consisting of Jennifer Kren and Callum Shoosmith (Editorial Coordinators), Teagan Meadows (Production Coordinator) and Amie Mustill (Production Manager) for their attention to detail and conscientious work in making sure that *Proceedings B* runs efficiently and on time. As in previous years, I would especially like to thank the Publishing Editor of *Proceedings B,* Shalene Singh-Shepherd, for her continuing dedication to the journal and wise counsel. I am especially grateful to both Shalene and Jennifer for overseeing the implementation of DAPR and the use of data editors in an almost seamless fashion. From all of us at *Proceedings B* we wish you all a productive and healthy 2025.
